# Embracing goodbye: A personal reflection on virtual reality intervention in palliative care

**DOI:** 10.1017/S1478951525000161

**Published:** 2025-02-21

**Authors:** Olive Kit Ling WOO

**Affiliations:** Department of Psychology, The University of Hong Kong, Hong Kong, Hong Kong

As a clinical psychologist working in the palliative care unit of a public hospital, I have integrated virtual reality (VR) technology into my clinical work, offering over 400 patients with terminal illnesses in the past 5 years. Although recent systematic reviews (Huang et al. 2024; Martin et al. 2022; Mo et al. 2022; Moloney et al. 2023; Moutogiannis et al. 2023; Vasudevan et al. 2023) convergently support the feasibility and acceptability of VR interventions in symptom control, such as managing pain and anxiety, there remains limited focus on the use of VR for facilitating goodbyes during the final stage of life. This gap in the literature inspired me to explore the potential of VR intervention beyond symptom management that traditional intervention appears impossible to address. In this essay, I reflect on a clinical case that brought into focus the potential of VR in facilitating adaptive coping during anticipatory grief of family members. This case not only shaped my understanding of the potential of VR intervention but also potentiated my personal and professional growth as a clinician.

## A clinical case of saying goodbye

Mr. L., an 89-year-old man with terminal cancer, was referred to our clinical psychology service in the final stage of his life. He was unconscious and unresponsive, his frail body tethered to the hospital bed. His family, desperate to honor his final wishes, requested support from the medical team. They had hoped to display cherished family photos in his ward using a projector – something that was not available in the inpatient setting.

When I met Mr. L.’s daughter, her face betrayed frustration and sorrow. She shared that her father’s last wish had been to return home and spend time with his family. Her words intertwined with sense of helplessness I often recognize in families presenting with anticipatory grief. I gently suggested the possibility of a VR intervention. I offered to lend the family a VR camera, so they could record their home environment for Mr. L. to view if his condition improved. The daughter gladly agreed.

The family, inspired by the suggestion, came together during the Chinese Mid-Autumn Festival – a holiday deeply rooted in themes of family reunion and gratitude. They not only filmed the home but also recorded heartfelt messages for Mr. L. Each family member spoke directly to Mr. L. through the VR camera, expressing gratitude and recounting how he had influenced their lives. It became not just a video but a vivid capture of love and warmth.

Unfortunately, Mr. L.’s condition did not allow for the video to be shown to him. His daughter, however, did not ask whether the video could be played, likely understanding the impossibility of the situation. Instead, she requested a soft copy of the recording for the family’s own keeping.

## Reflections on the journey

This interactive moment of the family has resonated with me, not only because of the potential positive outcome VR technology can bring about but also lessons it taught me about finding meaning amid helplessness with the help of this adjunct therapeutic tool.

### Personal growth: finding meaning amid helplessness

Clinicians providing palliative care often encounters emotional toll of witnessing patients and families confront the inevitability of death, or even experience emotional burnout (Dijxhoorn et al. 2023; Sapeta et al. 2022). As a clinical psychologist in the palliative care setting, I am not an exception. Sense of helplessness can creep into my clinical work, particularly when goodbyes are filled with pain and anticipatory grief. The use of VR in this case, however, potentiates support for the family when experiencing anticipating grief. Traditionally, psychology services focus on facilitating verbal conversations, emotional processing, and bereavement preparation. However, VR opened a door to something different – a way to help families actively engage in their grief, transforming passive waiting into a process of emotional and meaningful expression.

Though a traditional 2D camera could also record such messages, the immersive nature of VR is what sets it apart. Recent research conducted by Niki et al. (2024) has similarly shown that VR’s immersive nature allows hospitalized patients to effectively communicate with family through smooth conversation. Even though my patient, Mr. L., could not view the video, the process itself provided his family with a sense of agency and comfort. For me, it reaffirmed the power of innovation in fostering resilience – not just for patients and families but also for clinicians – that I can explore every possible means to assist the family in face of patient’s imminent death. It reminded me of the words of Dame Cicely Saunders, the founder of palliative care: “We will do all we can not only to help you die peacefully, but also to live until you die.” VR intervention allows me to support the family with a realization that I can do something more for them.

### Professional growth: VR as a psychological tool

The experience also reshaped my professional approach to care. While VR is often seen as a technological innovation, I have come to view it as a psychological tool with unlimited therapeutic potential. In this case, VR was not merely a medium; it has high potential to be a therapeutic intervention in itself. It can act as an adjunct tool in facilitate adaptive coping amid anticipatory grief, providing an opportunity to deliver goodbyes or gratitude amid patient’s frail condition. Accordingly, for therapists who deliver VR interventions are advised to be trained not only in the technical aspects of VR technology but also in understanding its psychological impact.

## Implications for clinical practice and research

This experience has several implications for both clinical practice and research. Recent studies have shown that digital intervention such as videoconference and VR experience can lead to improved emotional outcomes and foster deeper connections within families (Fernandez-Bueno et al. 2024; Kokorelias et al. 2024). The current case unveils that VR interventions can be further explored to allow family members to film personalized videos, which can mitigate the usual challenges of high costs and low degrees of personalization typically associated with tailored-made materials (Coelho et al. 2020; Huang and Yang 2022). Further research is advised on investigating the benefit of such approach to both patients and caregivers who create the videos.

Research-wise, more robust studies are needed to evaluate the efficacy of VR interventions in palliative care settings, not only on symptom management but also on anticipatory grief. As literature has shown VR mindfulness intervention can reduce burnout levels and improved work engagement among healthcare professionals (Ferrer Costa et al. 2024), further investigation is suggested on the benefits that such novel VR interventions in the current case can bring to therapists, such as enhancing self-efficacy, reducing feelings of helplessness, and ultimately minimizing burnout.

## Conclusion

This clinical case taught me that there is no single “right” way to say goodbye. Whether through words, actions, or innovative tools like VR, the act of parting can be an opportunity for connection, healing, and growth. As a psychologist, I have often felt that one of my roles is to hold space for grief.But this experience showed me that I can do much more – I can help families choose a more proactive and adaptive way through innovative technology during the most suffocating moments. VR, in this context, is not just a technological innovation; it appears to be a bridge between worlds, offering patients and families a way to connect when time and space seem insurmountable. It is a reminder that even in the most challenging moments, there is room for creativity, resilience, and hope (Frankl 2006). Mr. L.’s story is one of many, but it is one I will carry with me as I continue to explore the intersection of technology and humanity in palliative care. For in the art of saying goodbye, we often discover the essence of what it means to live.
